# Role of Gut Microbiome in Autism Spectrum Disorder and Its Therapeutic Regulation

**DOI:** 10.3389/fcimb.2022.915701

**Published:** 2022-07-22

**Authors:** Masuma Afrin Taniya, Hea-Jong Chung, Abdullah Al Mamun, Safaet Alam, Md. Abdul Aziz, Nazim Uddin Emon, Md. Minarul Islam, Seong-T shool Hong, Bristy Rani Podder, Anjuman Ara Mimi, Suzia Aktar Suchi, Jian Xiao

**Affiliations:** ^1^ Department of Life Sciences, School of Environment and Life Science, Independent University, Dhaka, Bangladesh; ^2^ Gwanju Center, Korea Basic Science Institute, Gwanju, South Korea; ^3^ Molecular Pharmacology Research Center, School of Pharmaceutical Sciences, Wenzhou Medical University, Wenzhou, China; ^4^ Drugs and Toxins Research Division, BCSIR Laboratories, Rajshahi, Bangladesh Council of Scientific and Industrial Research, Rajshahi, Bangladesh; ^5^ Department of Pharmacy, Faculty of Pharmacy and Health Sciences, State University of Bangladesh, Dhaka, Bangladesh; ^6^ Department of Pharmacy, Faculty of Science and Engineering, International Islamic University Chittagong, Chattogram, Bangladesh; ^7^ Department of Biomedical Sciences and Institute for Medical Science, Jeonbuk National University Medical School, Jeonju, South Korea; ^8^ Department of Biochemistry and Molecular Biology, University of Dhaka, Dhaka, Bangladesh; ^9^ Department of Pharmacy, Faculty of Allied Health Sciences, Daffodil International University, Dhaka, Bangladesh; ^10^ Department of Pharmacy, College of Pharmacy, Chosun University, Gwangju, South Korea; ^11^ Department of Hand Surgery and Peripheral Neurosurgery, The First Affiliated Hospital of Wenzhou Medical University, Wenzhou, China

**Keywords:** autism spectrum disorder, microbiota–gut–brain axis, gut microbiota, dietary fibers, microbial therapeutics

## Abstract

Autism spectrum disorder (ASD) is a neurological disorder that affects normal brain development. The recent finding of the microbiota–gut–brain axis indicates the bidirectional connection between our gut and brain, demonstrating that gut microbiota can influence many neurological disorders such as autism. Most autistic patients suffer from gastrointestinal (GI) symptoms. Many studies have shown that early colonization, mode of delivery, and antibiotic usage significantly affect the gut microbiome and the onset of autism. Microbial fermentation of plant-based fiber can produce different types of short-chain fatty acid (SCFA) that may have a beneficial or detrimental effect on the gut and neurological development of autistic patients. Several comprehensive studies of the gut microbiome and microbiota–gut–brain axis help to understand the mechanism that leads to the onset of neurological disorders and find possible treatments for autism. This review integrates the findings of recent years on the gut microbiota and ASD association, mainly focusing on the characterization of specific microbiota that leads to ASD and addressing potential therapeutic interventions to restore a healthy balance of gut microbiome composition that can treat autism-associated symptoms.

## Introduction

Concerns over autism spectrum disorder (ASD) are alarming, as many people are being diagnosed every year. Statistics report that ASD affects 1 in 68 people worldwide (DeFilippis and Wagner, 2016; Li et al., 2017). ASD is a highly prevalent neurodevelopmental disorder (Tsai et al., 2012) that affects normal brain development and is characterized by poor communication skills, poor reasoning, and repetitive and obstructive behavioral patterns (Louis, 2012; DeFilippis and Wagner, 2016; Liu et al., 2019). Doctors try to alleviate these conditions through behavioral therapies and specific therapeutic interventions (Tsai, 2000; Levy et al., 2003; Wong and Smith, 2006). They often administer drugs such as Aripiprazole, Escitalopram, antidepressants, and drugs that affect the concentration of a neurotransmitter or chemical messenger called serotonin in the brain (Shapiro et al., 2003; de Bartolomeis et al., 2015). Selective serotonin uptake inhibitors (SSRIs) may improve the communication, social skills, and adaptability of the patients. Previous studies have suggested that therapeutic interventions may also cause hyperactivity and aggression and side effects such as vomiting, irritability, increased appetites, weight gain, and sedation (Soorya et al., 2008; DeFilippis and Wagner, 2016). Scientists have been trying to explore the molecular mechanisms behind the pathology of ASD to facilitate alternative treatments. Several evidence suggests that genetic factors such as chromosomal abnormalities and environmental factors like diet and stress are involved in the pathogenesis and advancement of ASD (Matsuzaki et al., 2012; Ma et al., 2019). Over many years, parents have reported that their children diagnosed with ASD also suffer from gastrointestinal (GI) symptoms like constipation, abdominal pain, diarrhea, and vomiting (Soorya et al., 2008; DeFilippis and Wagner, 2016; Li et al., 2017; Warner, 2018; Babinská et al., 2022) Interestingly, accumulating research has demonstrated the gut–brain axis or multiple biochemical signaling pathways that take place between the gastrointestinal tract (GI tract) and the central nervous system (CNS), and its possible association with ASD (Kim et al., 2018). Mounting evidence explains that gut microbial dysbiosis is implicated in the pathogenesis of multiple diseases, including inflammatory bowel disease (IBS), coeliac disease (CD), allergy, asthma, metabolic syndrome, cardiovascular disease, obesity, and ASD (Carding et al., 2015; Kumar et al., 2017; Noor et al., 2021). This mini-review focuses on the association between ASD and the gut microbial population. We also addressed the impact of unregulated antibiotic usage on the gut microbiome, the pathogenesis involved, and various potential microbial therapeutics such as fecal microbiota transplantation (FMT) for treating autistic patients.

## Microbiota–Gut–Brain Axis

**Figure 1:**
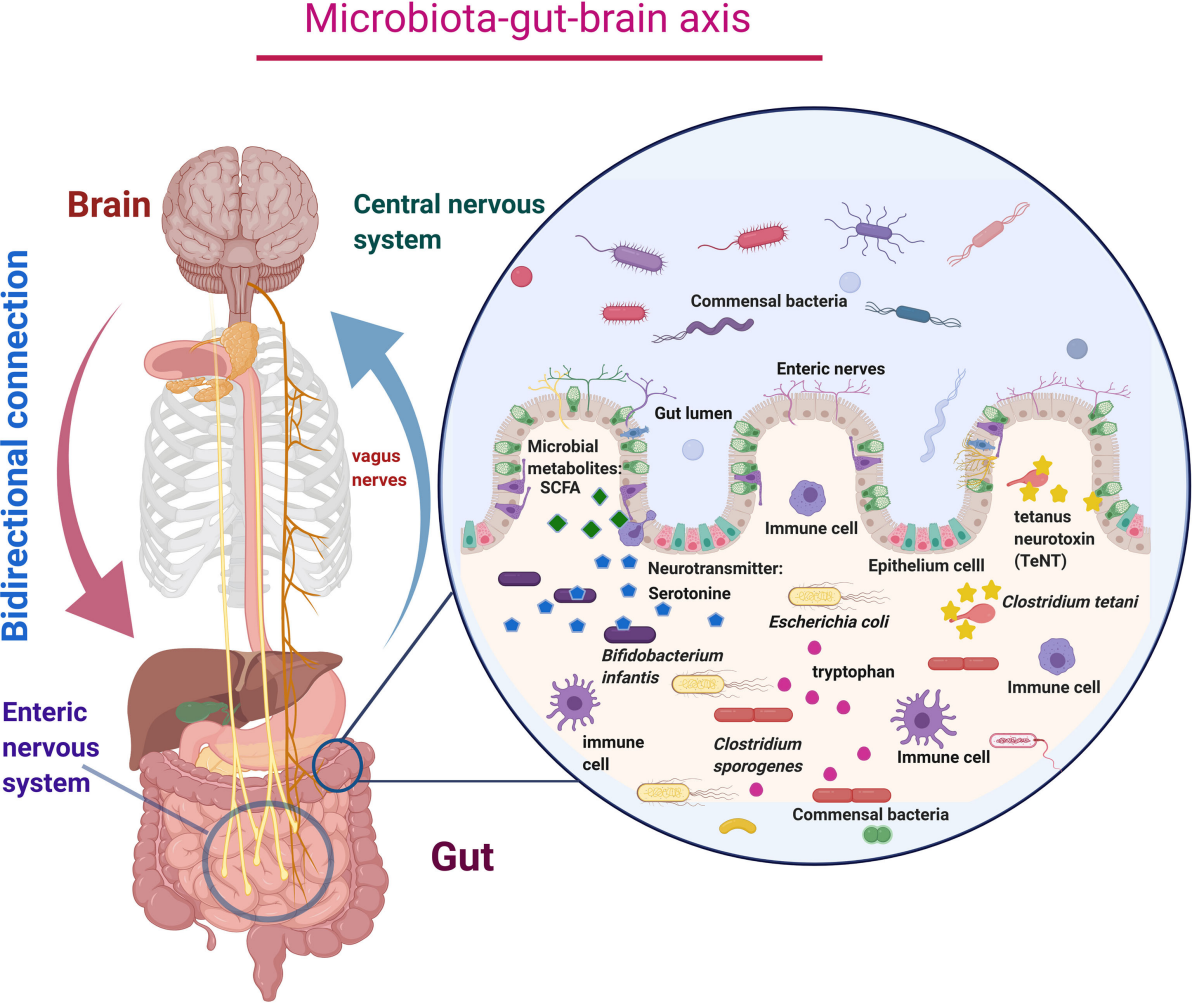
Schematic diagram of microbiota–gut–brain axis represents the bidirectional connection between the central nervous system (CNS) and enteric nervous system (ENS) *via* the vagus nerves, which carry neurotransmitters like serotonin, tetanus neurotoxin, and microbial metabolites like SCFA produced by the microbial action. Millions of immune cells in the enteric nervous system cause immune-mediated functions and keep healthy microbial colonization.

Recent studies on the gut–brain axis suggested that the gut contains millions of nerve cells, eventually forming an extensive network called the enteric nervous system (ENS). This enteric nervous system is also considered our second brain (Gershon, 1999). The ENS and the central nervous system (CNS) are mainly connected *via* the vagus nerve and form the gut–brain axis (Goyal and Hirano, 1996; Flannery et al., 2019). The communications within the gut–brain axis occur through the autonomic nervous system, enteric nervous system, neurotransmitters, hormones, and immune responses (**Figure 1**) (Cryan and Dinan, 2012). Neurotransmitters produced in the gut influence our emotions by regulating the gut–brain axis. Around 90% of neurotransmitters, such as serotonin, are produced in the gut (Mohammad-Zadeh et al., 2008; Warner, 2018). The human gut microbiome consists of trillions of bacterial cells, which is 10-fold more significant than human body cells (Chauhan, 2017; Kumar et al., 2018). The average human gut contains 1 kg of diverse groups of bacteria that mostly perform beneficial activities often involved in metabolites production and transportation and maintenance of gut homeostasis (Dinan et al., 2015; Kumar Mondal et al., 2017; Li et al., 2017). Earlier, the majority of microbiota cannot be classified by traditional cultural techniques. Researchers have overcome this limitation by using next-generation sequencing technology and metagenomic sequencing to identify unculturable microorganisms (Wang et al., 2015; Chauhan et al., 2017). Our intestines harbor numerous beneficial bacteria that generate many neurotransmitters and active metabolites in the gut by utilizing the consumed foods. Essential amino acid tryptophan found in food acts as a precursor of a neurotransmitter called serotonin. Over 1%–2% of dietary tryptophan is converted into serotonin (Jennis et al., 2017). Beneficial gut bacteria such as *Bifidobacterium infantis* convert tryptophan into serotonin, regulating emotions and behavior (Desbonnet et al., 2008)*. Clostridium sporogenes* increases the production of tryptophan metabolites called indole-3-propionic acid (IPA), a bioactive molecule that increases the production of antioxidant and neuro-protectant molecules inside the gut (**Table 1**) (Wikoff et al., 2009; Javier Díaz-García et al., 2020). The gut can also be invaded by various pathogenic microbes, leading to the development of gastrointestinal problems following the presence of *Clostridium bolteae* (Tsai et al., 2012; Rosenfeld, 2015). Interestingly, *Clostridium* bacteria in the colon indicate higher risk and severity of ASD (Soorya et al., 2008; Rosenfeld, 2015). This specific strain of bacteria produces tetanus neurotoxin (TeNT), which passes through the vagus nerve to the CNS and blocks neurotransmitters by the proteolytic cleavage of synaptobrevin, a synaptic vesicle membrane protein, and precipitates a whole range of behavioral deficits. Li et al. have implied that the presence of *Clostridium tetani* can be used as an indicator for the ASD diagnosis (**Table 1**) (Li et al., 2019).

**Table 1:** Difference in the mode of delivery represents distinctive gut microbiota consortia, which produce metabolites and play essential roles in the human body.

Microorganisms (higher abundance)	Mode of delivery	Bacterial metabolites	Role of bacteria in the human body	References
*Enterococcus* spp. (*Enterococcus faecalis*, *Enterococcus faecium*)	C-section	Extracellular superoxide Hydrogen peroxide	DNA damage in colorectal cancer	(Zhu et al., 2013; Shao et al., 2019)
*Klebsiella* spp. (*Klebsiella pneumoniae*, *Klebsiella oxytoca)*	C-section	Trimethylamine (TMA), serotonin, indole	Involve in the pathogenesis of cardiovascular disease through the gut microbiota-mediated pathway	(Shao et al., 2019; Averina et al., 2020., Kalnins et al., 2015)
*Bacteroides* spp. (*Bacteroides thetaiotaomicron*)	Vaginal	Succinate	Provide nutrients for the growth of other gut microbiota, maintain gut homeostasis	(Mitsou et al., 2008; Kabeerdoss et al., 2013; Shao et al., 2019; Ikeyama et al., 2020; Yadav and Chauhan, 2020)
*Bifidobacterium* spp. (*Bifidobacterium infantis*)	Vaginal	Serotonin, Lactate and acetate	Regulates emotions and behavior; Maintain gut homeostasis, produce vitamins and antimicrobial substances, and regulates the host immune system	(Mitsou et al., 2008; Kabeerdoss et al., 2013; Devika and Raman, 2019)
*Lactobacillus* spp. (*Lactococcus lactis*, *Lactobacillus nodensis*, *Lactobacillus vini*, *Lactobacillus paraplantarum*)	Vaginal	L-glutamate, trimethylamine, serotonin, imidazolone, propionate, and taurine	Improve the brain function and elevate mood	(Mitsou et al., 2008; Kabeerdoss et al., 2013; Averina et al., 2020; Giri and Sharma, 2022)
*Clostridium* spp. (*Clostridium sporogenes*, *Clostridium bolteae*, *Clostridium tetani*, *Clostridium perfringens*, *Clostridium difficile*)	C-section	Indole-3-propionic acid (IPA), Bacteriocins, Short-chain fatty acid (butyrate)	Increases the production of antioxidant and neuro-protectant molecules inside the gut; acts as a biomarker for ASD; inhibits the growth of other gut microbiota, promotes the growth or virulence of gut pathogens	(Penders et al., 2005; Penders et al., 2006; van Nimwegen et al., 2011; Pandey et al., 2012)
*Prevotella* spp. (*Prevotella intestinalis*)	Vaginal	Taurine, histamine, polyamines, and SCFAs	Regulate inflammatory receptors during transcription	(Dominguez-Bello et al., 2010; Iljazovic et al., 2020)
*Sneathia* spp. (*Sneathia amnii*)	Vaginal	Mucins, sialic acid, Polyamines	Causes bacterial vaginosis	(Dominguez-Bello et al., 2010; Łaniewski and Herbst-Kralovetz, 2021)
*Sutterella* spp.	C- section	Acetate	Involve in the pathogenesis of ASD	(Ding et al., 2016; Toscano et al., 2017; Sun et al., 2020)
*Bilophila* spp. (*Bilophila wadsworthia*)	Vaginal	Indole	Involve in the pathogenesis of cardiovascular disease through the gut microbiota-mediated pathway	(Kivenson and Giovannoni, 2020; Chen et al., 2021)
*Achromobacter* spp. (*Achromobacter liguefaciens*)	Vaginal and C-section	Indole	Increases the degradation of tryptophan, leads to the build-up of neurotransmitters in the brain of the autistic patient, and blocks the efflux transporter in the blood–brain–barrier (BBB)	(Toscano et al., 2017; Srikantha and Mohajeri, 2019; Chen et al., 2021)
*Bacillus* spp. (*Bacillus subtilis*)	Vaginal	Serotonin, dopamine, and noradrenaline	Regulates brain function and elevates mood	(Srikantha and Mohajeri, 2019; Averina et al., 2020; Chen et al., 2021)
*Ruminococcaceae* spp.	C-section and Vaginal	Propionic acid and L-glutamate	Leads to behavioral and physiological deficits in ASD	(Toscano et al., 2017; Srikantha and Mohajeri, 2019; Averina et al., 2020; Chen et al., 2021)
*Faecalibacterium* spp. (*Faecalibacterium prausnitzii*)	Vaginal	Butyric acid	Prevents inflammatory response in the gut, promotes memory formation, and enhances neuronal plasticity through epigenetic regulation	(Srikantha and Mohajeri, 2019; Averina et al., 2020; Chen et al., 2021)
*Corynebacterium* spp. (*Corynebacterium glutamicum*)	C-section	L-glutamate, phenylalanine, and tryptophan	Tryptophan acts as a precursor of serotonin, which improves mood; phenylalanine is considered a biomarker for depression-like symptom	(Dominguez-Bello et al., 2010; Srikantha and Mohajeri, 2019; Averina et al., 2020)
*Pseudomonas* spp. (*Pseudomonas putida*)	C-section	Serotonin	Regulates brain function and elevates mood	(Adlerberth et al., 2006; Dominguez-Bello et al., 2010; Hesla et al., 2014; Dogra et al., 2015; Srikantha and Mohajeri, 2019; Averina et al., 2020)
*Staphylococcus* spp. (*Staphylococcus aureus*)	C-section and Vaginal	Serotonin	Regulates brain function and elevates mood	(Dominguez-Bello et al., 2010; Toscano et al., 2017; Srikantha and Mohajeri, 2019; Averina et al., 2020; Kim et al., 2020)
*Akkermansia* spp. (*Akkermansia muciniphilia*)	C-section	Propionic acid and acetic acid	Leads to behavioral and physiological deficits in ASD	(Toscano et al., 2017; Srikantha and Mohajeri, 2019; Averina et al., 2020)
*Streptococcus* spp. (*Staphylococcus epidermidis*, *Streptococcus parasanguinis*)	C-section	Glutathione	Protects tissues from oxidative stress	(Averina et al., 2020; Chen et al., 2021)
*Coprococcus* spp. (*Coprococcus comes*, *Coprococcus eutactus*, *Coprococcus catus*)	Vaginal	Butyric acid and propionic acid	Promotes memory formation and enhances neuronal plasticity through epigenetic regulation	(Toscano et al., 2017; Srikantha and Mohajeri, 2019; Averina et al., 2020; Chen et al., 2021)
*Citrobacter* spp. (*Citrobacter koseri*)	C-section	Indole	Increases the degradation of tryptophan, leads to the build-up of neurotransmitters in the brain in ASD, and blocks the efflux transporter in the blood–brain barrier (BBB)	(Srikantha and Mohajeri, 2019; Averina et al., 2020; Chen et al., 2021)
*Haemophilus* spp. (*Haemophilus influenza*)	C-section	Indole	Increases the degradation of tryptophan, leads to the build-up of neurotransmitters in the brain of the autistic patient, and blocks the efflux transporter in the blood–brain barrier (BBB)	(Srikantha and Mohajeri, 2019; Averina et al., 2020; Chen et al., 2021)
*Eubacterium* spp. (*Eubacterium hallii*, *Eubacterium rectale*, *Eubacterium rectale*)	Vaginal	Indole, propionic acid, and butyric acid	Promotes memory formation and enhances neuronal plasticity through epigenetic regulation	(Toscano et al., 2017; Srikantha and Mohajeri, 2019; Averina et al., 2020; Chen et al., 2021)

## Effect of Early Colonization on ASD

Numerous evidence explains that microbial colonization starts during prenatal development inside the mother’s womb, in the placenta (Stout et al., 2013), and amniotic fluid (Collado et al., 2016). Bacteria colonization in the maternal–fetal unit can have beneficial or adverse effects on pregnancy and/or fetal development (Taddei et al., 2018). Colonization of some bacterial species such as *Lactobacillus*, *Enterococcus*, *Streptococcus*, *Peptostreptococcus*, *Corynebacterium*, *Escherichia*, and *Staphylococcus in infants* passed from the breast milk of lactating mothers (Noor et al., 2020). Pioneer microbial colonization inside the GI tract of preterm infants begins at birth. After 1 year in which a unique and complex microbiota develops, the composition of the microbiota stabilizes around the age of 2–3 years. The intrauterine period during pregnancy and postnatal period can provide a critical window for infant microbiome development, which has lifelong ramifications on overall health (Taddei et al., 2018; Warner, 2018; Lee, 2019). Interestingly, the brain of neonates also grows from 36% to approximately 90% of its future adult volume until the age of 2. Thus, establishing a healthy microbial composition falls into the same critical time for brain development (Srikantha and Mohajeri, 2019). A study with rat models has demonstrated that maternal separation from infant rats daily for 3 h from day 2 to 12 postnatal led to microbial dysbiosis. After being exposed to chronic stress, there was an elevation in the abundance of *Bacteroides* and *Clostridium* in rats and an increase in immune cells such as pro-inflammatory cytokines and chemokines (IL-6 and CCL2) (Forssberg, 2019).

## Maternal Microbiota in the Early Gestation Period and Their Effect on Fetal Neurodevelopment

The interval between conception and gestation is a crucial period for fetal neurodevelopment. During this time, several factors, such as an unhealthy diet (Buffington et al., 2016), microbial infection (Kim et al., 2017), and metabolic stress (Jašarević et al., 2018), can lead to maternal microbiome dysbiosis that may influence the abnormal neurological development of offspring, leading to lifelong behavioral deficits (Vuong et al., 2017).

The study conducted by Vuong et al. in a mice model of three categories namely, antibiotic-treated (ABX) embryos and specific pathogen-free (SP) and germ-free (GF) mice mothers, demonstrated that colonization of *Clostridia*-dominant spore-forming bacteria in maternal microbiota leads to the reduction of fetal brain gene expression and thalamocortical axonogenesis connecting the thalamus and the cerebral cortex and impaired outgrowth of thalamic axons (Yaguchi et al., 2014). Previous studies have shown that spore-forming *Clostridia* species downregulate NTNG1 expression and minimize netrin-G1a+ thalamocortical axons, leading to the impairment of thalamic axon outgrowth (Yaguchi et al., 2014). Vuang and co-workers have demonstrated that colonization of Bacteroidetes (BD) in mother mice may significantly enhance netrin-G1a+ thalamocortical axons (Vuong et al., 2020). The healthy microbial composition inside the maternal mice can be restored *via* fecal transplantation, increasing the relative abundance of Bacteroides species by 95%. The relative abundance of *Clostridium difficile* was reduced to 5% (Botta et al., 2020; Vuong et al., 2020).

In a mother’s intestinal tract, a specific bacterial consortium produces metabolites that influence the development of the fetal brain. Further experiments showed that the maternal gut microbiota promotes healthy brain development by regulating biochemical profiles and selecting metabolites in the fetal brains of developing offspring. During pregnancy, the gut microbiota modulates the bioavailability of many biochemicals, nutrients, and growth factors that circulate in the maternal blood, supporting offspring growth and proper fetal brain development. After analyzing the molecules in the mothers’ blood and their offspring’s blood and brain, they found that the levels of specific metabolites were reduced in GF and ABX pregnant mothers (Botta et al., 2020). SP mice, ABX, and GF mice were exposed to thalamic explants. The result showed that an increase in metabolites, such as trimethylamine-N-oxide (TMAO) (Molnár et al., 2012), N,N,N-trimethyl-5-aminovalerate (TMAV), imidazole propionate (IP), 3-indoxyl sulfate (3-IS), and hippurate (HIP), in embryos of SP mice compared to ABX and GF mice, may increase axon numbers *via* thalamocortical axonogenesis (Molnár et al., 2012; Vuong et al., 2020). Restoration of these metabolites into the microbiota-deficient mice mothers can potentially prevent the abnormalities in the development of neuronal connections and improve the behavior of the offspring. However, there might be increases in relationships with other neurons in the presence of the metabolites. Further studies are highly required to implement and analyze the result of this study in human maternal gut microbiota during pregnancy (Vuong et al., 2020).

### Effect of Mode of Delivery on ASD

Multiple studies have shown that babies born *via* vaginal delivery have more composition of healthy bacteria than the babies born *via* cesarean section delivery (Groer et al., 2015; Rosenfeld, 2015). Babies born *via* standard (vaginal) delivery are exposed to the mother’s vaginal bacteria, whereas children born *via* C-section delivery are remarkably exposed to the mother’s skin flora and environmental microorganisms. During the prenatal period, the fetus’ gut acquires a complex mixture of bacteria from mothers *via* breastfeeding exposure to vaginal and enteric microorganisms (Groer et al., 2015). At birth, the fetus’ microbiota is almost identical to the mother’s microbiome (Li et al., 2017; Forssberg, 2019). At this stage, the abundance of lactobacilli became the pioneer colonizer of the infant’s gut. The subsequent colonization of aerobic or facultative anaerobic bacteria like enterobacteria, enterococci, and staphylococci are found. The colonization of these bacteria uses oxygen for their growth and makes a suitable habitat for the colonization of anaerobes, including *Bifidobacterium*, *Clostridium*, and *Bacteroides*, and later prominent phyla such as Firmicutes and *Bacteroides*, and *Verrucomicrobia*; less dominant microbiota such as *Proteobacteria* and aerobic Gram-negative bacteria are developing inside the gut of adult human (Palmer et al., 2007; Shao et al., 2019). On the other hand, babies born *via* C-section were dominated by *Enterococcus faecalis*, *Enterococcus faecium*, *Staphylococcus epidermidis*, *Streptococcus parasanguinis*, *Klebsiella oxytoca*, *Klebsiella pneumoniae*, and *Clostridium* spp. (*Clostridium perfringens*, *Clostridium difficile*) (**Table 1**); these microbes are predominant at the mother’s skin surface and hospital environment (Stewart et al., 2015; Lax et al., 2017; Shao et al., 2019). Growing evidence displays that a globally increased cesarean section (CS) can also alter gut microbial composition in early infancy and might delay neurological adaptation in infants (Rosenfeld, 2015; Chen et al., 2017). Surprisingly, a current meta-analysis study has demonstrated that a child born *via* C-section delivery has a 23% risk of developing ASD compared with a child born *via* vaginal delivery (O’Mahony et al., 2009; Curran et al., 2014). In a most significant multi-national population-based study, which consisted of 5 million births from Norway, Sweden, Denmark, Finland, and Western Australia, each participant was observed for 36–42 weeks. More than 31,000 cases of ASD were confirmed, which supported the hypothesis that birth delivery by C-section possesses a higher risk of ASD than vaginal delivery (Yip et al., 2016).

### Effect of Antibiotics on ASD

From birth until about the age of 3, a human’s microbiome starts to develop. Using antibiotics during these formative years can disrupt the development of immune-mediated, metabolic, and neurological diseases (Arrieta et al., 2014). Antibiotics significantly alter the microbial composition by inhibiting the growth of pathogenic microbes (Ahmed et al., 2013a; Ahmed et al., 2013b; Ahmed et al., 2014). Early and uncontrolled doses of antibiotics may lead to loss of predominant microbial phyla, loss of diversity, change in metabolic activity, and colonization of pathogens (Bourassa et al., 2016; Chen et al., 2017). Antibiotic treatment during the 1–2 years of life can crucially impact the maturation of the immune system and have a detrimental effect on typical microbiota establishment with severe long-term consequences, such as inflammation, immune dysregulation, allergies, infections, and GI diseases such as Crohn’s, inflammatory bowel disease (IBD), constipation, and diarrhea (Jernberg et al., 2007; Yang et al., 2009; Ubeda et al., 2010; Ni et al., 2017; Warner, 2018). Further cohort studies performed using antibiotics during infancy and early childhood showed significant alterations in gut microbiota, which could be directly responsible for turning on or off specific genes. Early use and overuse of antibiotics lead to microbial dysbiosis and may turn on the autism gene. This affects the gut–brain axis by causing epigenetic modification, which potentially facilitates the pathogenesis of ASD (Eshraghi et al., 2018).

Early colonization of beneficial gut bacteria during infancy is essential for maintaining gut homeostasis. Using antibiotics in that period can increase gut microbiome dysbiosis inside the infant gastrointestinal tract (Eshraghi et al., 2018). A recent study showed that 34.5% of autistic children were exposed to extensive and repeated broad-spectrum antibiotic treatments, which was more than six courses compared to a control group with more than six courses. Further investigations are required to understand the impact of antibiotics overuse during the first years of life on the gut–brain axis and its association with other health outcomes such as autism in later years (Parracho et al., 2005).

### Importance of Dietary Fibers

According to recent studies, dietary fiber significantly increases microbial diversity. In a dietary intervention, subjects were provided dietary fiber. After 2 weeks, there was a significant change in microbial diversity observed among 20% of the fiber consumers, with an increase in the abundance of fiber degrading beneficial bacteria such as *Prevotellacopri* (Johnson et al., 2021).

Microbial fermentation of dietary fibers by gastrointestinal bacteria, such as Firmicutes, *Clostridia*, *Bacteroides*, and *Desulfovibrio*, produce short-chain fatty acids (SCFAs) butyrate, acetate, and propionate (MacFabe, 2012; Yadav et al., 2017; Yadav et al., 2020). SCFA induces pyruvate dehydrogenase kinase (PDK), leading to the inactivation of the pyruvate dehydrogenase complex. As a result, cells cannot produce sufficient energy due to the cessation of pyruvate to acetyl-CoA conversion (Kumar et al., 2020). To meet the energy requirement of the cell, colonocytes produce acetyl-CoA *via* β-oxidation of the SCFA. As the oxidative respiration increases in the cell, the oxygen concentration decreases, resulting in the stabilization of hypoxia-inducible factor-1α (HIF-1α). HIF-1α is a regulator of oxygen homeostasis controlled by the oxygen concentration inside the intestinal lumen (Kumar et al., 2020). Kumar et al. have reported that hypoxia-inducible factor 1-alpha (HIF1α) prevents the transportation of gut microbiota and protects the microorganisms from host immune response (Kumar et al., 2020).

The importance of dietary fibers and their impact on the CNS has been demonstrated in several epidemiological studies (Cenit et al., 2017). SCFA is a neuroactive compound produced by gastrointestinal bacteria, which leads to alteration of metabolic and immune system function and regulates the gene expression (MacFabe, 2012; Al-Asmakh et al., 2012; MacFabe, 2015). Butyrate, one of the short-chain fatty acids (SCFAs) produced, can profoundly improve the function of the CNS by inhibiting histone deacetylases (HDACs) (O’Mahony et al., 2009; Ding et al., 2016; Oriach et al., 2016; Lee, 2019). Histone deacetylases inhibition is necessary because it does not allow acetylation of histone protein, which eventually inhibits loosen up of the chromatin for acetyl groups to be added to N-terminal L-lysine residues by histone acetyltransferases (HATs) for transcription. Lack of histone acetyltransferases activity leads to transcriptional dysfunction, potentially causing many neurodegenerative diseases (Sleiman et al., 2009). HDAC inhibitors such as sodium butyrate (NaB) have become very promising and attractive therapeutic candidates for their ability to increase histone acetylation and promote the expression of genes (Sealy, 1978). Conversely, a recent experiment showed that propionate injection inside the intracerebroventricular during development results in autistic-like behavior, and the same results were observed in a rodent model of autism (MacFabe et al., 2008; Bourassa et al., 2016). Excessive propionate can impact CNS function by crossing the gut–brain and blood–brain barriers, causing neuroinflammatory responses and behavioral alterations, leading to aggravation of neurodegenerative conditions like autism spectrum disorder (Macfabe et al., 2007; Wang et al., 2012). SCFAs can be both beneficial and detrimental to neurodegenerative disorders. Recent studies demonstrated that children with ASD have increased production of SCFAs, mainly indicated by the alteration in the fermentation of dietary fiber and abundance of SCFAs in ASD children compared to children without ASD (Wang et al., 2014). Therefore, these microbial fermentation products can also act as a biomarker for detecting ASD (Valicenti-McDermott et al., 2006).

## Microbiome Therapeutics

### Additive Therapy

Based on years of research, it has been projected that antibiotics applied for treatment impose a threat to the human gut microbiome due to antibiotic resistance within microbes and lack of specificity and efficacy. To overcome these challenges in developing modern medicines and reduce the dependency on antibiotics (Ahmed et al., 2013b), microbial therapies are widely popularized for using beneficial microbes, naturally found in the human body. It has undergone several advancements like genetic engineering to enhance host–microbe interaction and increase treatment efficacy and effectiveness (Yadav and Chauhan, 2021).

In additive therapy, a cocktail of beneficial microbes is extracted from the healthy human body and introduced into fecal microbial transplantation (FMT), or through the consumption of probiotics (Yadav and Chauhan, 2021). Probiotics are non-pathogenic beneficial microorganisms (such as *Lactobacillus* and *Bifidobacterium*) introduced into the body to restore the healthy composition of the gut microbiome (Sandler et al., 2000; Umbrello and Esposito, 2016). In FMT, the patient suffering from a gastrointestinal disorder is subjected to complete restoration of the intestinal microbial consortium. This alternative approach to probiotics and antibiotic treatment consists of bacteria, archaea, fungi, and viruses (bacteriophage), regulating gut microbial homeostasis and the host immune system (Żebrowska et al., 2021). FMT therapy has been successfully implemented in several studies related to GI disorders like recurrent *Clostridium difficile* infections (rCDIs) and IBD, demonstrating effective results with satisfactory efficacy, and children showed significant improvement in GI symptoms (Żebrowska et al., 2021).

### Subtractive Therapy

In subtractive therapy, engineered bacteriocins and bacteriophages that exert promising antimicrobial activity are used to target pathogens in the gut without causing harm to other microbes in the ecosystem. This is a much better option than antibiotic usage, which eventually develops antibiotic resistance.

Bacteriocins are peptides synthesized in the ribosome, which target unwanted microbes in various ways: releasing toxins, cessation of respiratory mechanisms, disintegrating the membrane, and cell death (Yadav and Chauhan, 2021). One recent study presented that *Lactobacillus salivarius* strain UCC118 produces broad-spectrum bacteriocin called Abp118, which acts against the food-poisoning pathogen *Listeria monocytogenes* (Gillor et al., 2008). Another mice model study demonstrated that bacteriocins produced by *Lactobacillus casei* L26 LAFTI prevented the growth of specific strains of *E. coli* and *L. monocytogenes* and protected the intestinal wall. Probiotic strain *Lactobacillus johnsonii* LA1 releases a particular bacteriocin that inhibits the growth of ulcer-causing bacteria *Helicobacter pylori* (Gillor et al., 2008). Research has also shown the effective use of bacteriocins against *Clostridium*, *Enterococcus*, *Pediococcus*, *Lactobacillus*, and *Leuconostoc* (Yadav and Chauhan, 2021).

On the other hand, Bacteriophages are viruses that are engineered to infect specific bacteria and kill the bacterial host in the process. Due to the rise in antibiotic resistance, bacteriophages have been proven to be effective in tackling bacterial infections. However, research has shown that both bacteriophage and bacterial populations can coexist inside the gut of the mice model, which is why more research is required to determine the appropriate conditions for effectiveness in the bacteriophage therapy (Mimee et al., 2016).

### Modulatory Therapy

Conversely, modulatory therapy may significantly restore a healthy balance in the gut microbiome by changing the diet, exercise, and antibiotics. This favors the colonization of beneficial microbiota over pathogens (Yadav and Chauhan, 2021). A healthy diet plays a crucial role in shaping the gut microbiome composition, depending on the amount, type, and balance of the important dietary macronutrients (carbohydrates, proteins, and fats) including micronutrients such as vitamin D (Conlon and Bird, 2014). A study was conducted to demonstrate the influence of dietary carbohydrate intervention on the composition of intestinal microbiota among two groups of 171 rural African and 172 urban European children. For this particular study, these two groups were subjected to completely different types of dietary carbohydrates. The result showed higher dominance of Bacteroidetes (73%) in the microbiota of African children, whereas in the EU children, there was a 27% abundance of Bacteroidetes and 51% abundance of Firmicutes (De Filippo et al., 2010). Our gut comprises proteolytic bacteria such as *Bacteroides* species (especially *Bacteroides fragilis*) and *Clostridium perfringens*, propionibacteria, streptococci, bacilli, and staphylococci (Macfarlane et al., 1986), those that convert protein into nitrogen and amino acids (Scott et al., 2012). Studies investigating the influence of dietary fats consumption showed that high-fat consumption reduces the concentration of short-chain fatty acid (butyrate) and the abundance of bifidobacteria (Scott et al., 2012). However, a high-fat diet reduces intestinal inflammation but causes an increase in plasma markers of inflammation and lipopolysaccharide circulation (Cani et al., 2007).

In addition to internal and external factors affecting the efficacy of microbial therapy, exercise (an environmental factor) positively impacts the healthy composition of microbiota, which regulates gut homeostasis and maintains gut integrity. It enriches the microbial diversity leading to an increase in the Bacteroidetes–Firmicutes ratio, contributing to the reduction in metabolic disorders and gastrointestinal disorders by enhancing the production of bacterial metabolites like SCFA (Monda et al., 2017).

## Significance of Microbial Therapeutics for Autism: Animal Studies, Clinical Trials, and Their Limitations

Remarkably, up to 70% of children with ASD have impaired GI function (Gondalia et al., 2012). Clinicians working with such children increasingly assume a link between ASD and gut dysfunction (Shen, 2015; Li et al., 2017; Warner, 2018). Studies conducted at McMaster University suggested a strong connection between the microbiome and behavioral disorders in mice. The investigators used a gut bacteria makeover to turn anxious mice into extroverted mice by transferring gut microbes from the former to the latter and vice versa. Within 3 weeks after the transplant, the anxious mice became more sociable with prompt responsiveness, while extroverted mice became more nervous with delayed responsiveness (Sharon et al., 2019). This experiment suggested that gut microbiota dysbiosis may further facilitate the development of neurobehavioral disorders (Luna et al., 2016). However, studies examining connections between the gut microbiome and ASD in humans have begun recently.

A better understanding of the gut–brain axis and the roles of gut bacteria may lead to the treatment of neurological disorders, including ASD, with fewer side effects (Krajmalnik-Brown et al., 2015). The exciting findings on the existence of a so-called “microbiota–gut–brain axis” support the hypothesis that the gut microbiota could trigger neuropsychiatric symptoms in ASD patients (Valicenti-Mcdermottet et al., 2006; van De Sande et al., 2014). Doctors usually recommend a plant-based diet, prebiotics, and probiotics to ASD patients. Prebiotic is a non-digestible food ingredient, which increases the abundance of commensal bacteria in the gut (Groer et al., 2015; Liang et al., 2019).

In a recent case, doctors noticed that a child with symptoms of autism had large numbers of pathogenic bacteria like *Clostridium tetani* in his gut (Shaw, 2010). Treatment with vancomycin caused immediate symptom reversal (Bolte, 1998). Treatment with anti-*Clostridium* antibiotics in some autistic children has significantly reduced abnormal behavior (Kang et al., 2017). However, vancomycin treatment has a significant drawback, as it is not selective to favorable or harmful bacteria. Thus, more research should be conducted to find more specific targeted antibiotics and discover an alternative approach to reduce the use of vancomycin as a part of pre-treatment in Microbiota Transfer Therapy (MTT) (Adams et al., 2019). Conversely, in the mouse model of autism, adding more symbiotic bacteria such as *B. fragilis* through fecal transplant profoundly ameliorates the ASD-associated symptoms (Mayer et al., 2014; Mangiola, 2016). Fecal transplant treatment can potentially treat ASD patients by consuming beneficial bacteria (Sanders et al., 2013; Xu, 2015; Li et al., 2017). It has received many criticisms for using a small sample group, no replication, and not considering possible other variables.

Over the years of intervention, significant improvements in GI and autism symptoms have been observed (Sandler et al., 2000). A clinical trial of MTT was performed on 18 participants for 2 years, consisting of antibiotics, bowel cleanses, a stomach-acid suppressant, and fecal microbiota transplant. After 2 years of treatment, significant improvement in GI- and ASD-related symptoms and elevation of relative abundances of *Bifidobacterium* and *Prevotella* were observed among all 18 participants. After the treatment, most participants experienced an increase in microbial diversity, even after 2 years of therapy in typicallydeveloping children (Adams et al., 2019). This study has demonstrated the long-term efficiency of MTT for ASD patients suffering from GI disorders (Kang et al., 2019), and permanent change in the gut ecosystem created a favored condition for the survival of new healthy microbes. However, the limitation of the study was that in certain bacteria, short reads, including conserved regions of bacterial 16S rRNA, were targeted for amplicon sequencing for the taxonomic classifications (Adams et al., 2019).

It is essential to address that MTT has significant drawbacks, and there are chances that potentially life-threatening pathogens from the donor might invade the recipient’s intestine during the treatment. In a particular incident, an immunocompromised patient suffered from severe illness, who eventually died after receiving multi-drug-resistant bacteria during microbiota transplantation (Adams et al., 2019). To avoid such an incident, proper microbial screening of the donor should be performed to identify multi-drug-resistant organisms.

## Conclusions, Current Challenges, and Future Perspectives

Recapitulating all mounting evidence elucidates the association between the gut–brain axis and ASD (Sanders et al., 2013). Several factors leading to ASD were identified, mainly early colonization on infant health development and the effect of early microbiota dysbiosis in the gestation period, mode of delivery, uncontrolled usage of antibiotics, and stress. These factors eventually lead to gut microbiome dysbiosis and colonization of pathogenic microbes, which impact the CNS function by the production of neurotoxins. The presence of these pathogenic bacteria, such as *Clostridium* found in the colon of children, shows the possibility of developing ASD. Studies also indicated the importance of two SCFA produced from microbial formation of dietary fiber, including butyrate and propionate. Butyrate improves brain function by inhibiting histone deacetylases, whereas propionate impacts brain function, leading to changes in behavior and aggressiveness in ASD patients. According to many studies on the gut microbiome, MTT can potentially treat autism-like symptoms, including the restoration of healthy gut microbiome composition in the gestation period and early stage of infancy. Most studies have been conducted in experimental animal models. Some studies have small sample sizes, especially studies performed using human volunteers. The majority of them lacked randomization or control groups. Significant limitations in these studies eventually reduced the validity of the results. Recently, scientists have carried out several clinical trials to investigate the efficacy of MTT on children who have ASD, and the results obtained were satisfactory, showing remarkable improvement in GI symptoms with minimal adverse effects due to pre-treatment with vancomycin (Kang et al., 2017). An increasing number of news articles and science media are featuring the successes of microbiome research and its promising therapeutics regime. The general population is excited to learn that the gut microbiome is linked to the progression of various diseases and is also essential for healthy organ development. Based on the forecasted information regarding the success of microbial therapeutics in clinical trials, people are hopeful that this treatment will be a better alternative compared to conventional medicinal practices (Bik, 2016).

In 2019, the Food and Drug Administration (FDA) recognized microbial transplant therapy and labeled it “fast-track” for ASD treatment after observing the successful clinical trials using long-term microbial transplant therapy on autistic children (Adams et al., 2019). Further research should be conducted to determine whether findings in animal studies and clinical trials can be successfully implemented with similar efficacy rates in different populations of ASD patients across various geographical locations and exhibit promising results among the patients. Furthermore, more follow-up studies are required with accurate methodologies and optimized dosage of antibiotics and microbial suspensions to increase the efficacy of the treatment to support the validity, reliability, and precision of past research. In addition, genomic analysis of the gut microbiome of the donor and recipient should further define specific strains, better pathways, and regimens to provide optimum supplements and treatment in this particular population (Gondalia et al., 2012; Sanders et al., 2013; Xu, 2015; Li et al., 2019; Nitschke et al., 2020; Yadav and Chauhan, 2021). There is an increase in demand to find a substitute for microbial transplant therapy and reduce the dependency on the harness beneficial microbiota from a healthy human, which can be achieved by culturing a specific combination of bacteria for transplantation (Adams et al., 2019). Microbiome research has made much progress in recent years from animal studies to clinical trials, with satisfactory results and better efficacy in MTT treatment. In the next couple of years, FDA-approved pills, probiotics, and metabolites might be commercially available to establish a suitable bacterial composition to treat and regulate ASD. Our review investigations strongly imply exploring the underlying molecular mechanism of the gut microbiome in the pathogenesis and advancement of ASD and finding promising therapeutic agents/drugs that will deliver new hope for the treatment and management of ASD in the near future.

## Author Contributions

MAT, AAM, SA, and NUE: wrote the manuscript draft, illustrated figure, and table. MAT and AAM: designed the review. HJC and JX: conceptualized and supervised the study. MMI, STH, and MAA, BRP: performed in searching works of literature. AAM, MAT and SAS: checked the manuscript the draft. All authors finally revised the paper and approved for final submission.

## Funding

This research was funded by the Korea Basic Science Institute (KBSI), grant C220000, C280320 and C210500.

## Conflict of Interest

The authors declare that the research was conducted in the absence of any commercial or financial relationships that could be construed as a potential conflict of interest.

## Publisher’s Note

All claims expressed in this article are solely those of the authors and do not necessarily represent those of their affiliated organizations, or those of the publisher, the editors and the reviewers. Any product that may be evaluated in this article, or claim that may be made by its manufacturer, is not guaranteed or endorsed by the publisher.

## References

[B1] Żebrowska P. Łaczmańska I. Łaczmański Ł. (2021). Future Directions in Reducing Gastrointestinal Disorders in Children With ASD Using Fecal Microbiota Transplantation. Front. Cell. Infection Microbiol. 11. doi: 10.3389/fcimb.2021.630052 PMC7952982 33718277

[B2] Adams J. Borody T. Kang D. Khoruts A. Krajmalnik-Brown R. Sadowsky M. (2019). Microbiota Transplant Therapy and Autism: Lessons for the Clinic. Expert Rev. Gastroenterol. Hepatol. 13 (11), 1033–1037. doi: 10.1080/17474124.2019.1687293 31665947

[B3] Adlerberth I. Lindberg E. Åberg N. Hesselmar B. Saalman R. Strannegård I.. (2006). Reduced Enterobacterial and Increased Staphylococcal Colonization of the Infantile Bowel: An Effect of Hygienic Lifestyle? Pediatr. Res. 59 (1), 96–101. doi: 10.1203/01.pdr.0000191137.12774.b2 16380405

[B4] Ahmed V. Kumar M. Kumar J. Chauhan M. Chauhan N. (2013b). Nanogold/Polyaniline/Penicillin G Nanoconjugates: A Novel Nanomedicine. Int. J. Polymeric Materials And Polymeric Biomaterials 63 (2), 86–91. doi: 10.1080/00914037.2013.769252

[B5] Ahmed V. Kumar J. Kumar M. Chauhan M. Dahiya P. Chauhan N. (2014). Functionalised Iron Nanoparticle–Penicillin G Conjugates: A Novel Strategy to Combat the Rapid Emergence of β-Lactamase Resistance Among Infectious Micro-Organism. J. Exp. Nanoscience 10 (9), 718–728. doi: 10.1080/17458080.2014.881570

[B6] Ahmed V. Kumar J. Kumar M. Chauhan M. Vij M. Ganguli M.. (2013a). Synthesis, Characterization of Penicillin G Capped Silver Nanoconjugates to Combat β-Lactamase Resistance in Infectious Microorganism. J. Biotechnol. 163 (4), 419–424. doi: 10.1016/j.jbiotec.2012.12.002 23305990

[B7] Al-Asmakh M. Anuar F. Zadjali F. Rafter J. Pettersson S. (2012). Gut Microbial Communities Modulating Brain Development and Function. Gut Microbes 3 (4), 366–373. doi: 10.4161/gmic.21287 22743758 PMC3463494

[B8] Arrieta M. Stiemsma L. Amenyogbe N. Brown E. Finlay B. (2014). The Intestinal Microbiome in Early Life: Health and Disease. Front. In Immunol. 5. doi: 10.3389/fimmu.2014.00427 PMC4155789 25250028

[B9] Averina O. Zorkina Y. Yunes R. Kovtun A. Ushakova V. Morozova A.. (2020). Bacterial Metabolites of Human Gut Microbiota Correlating With Depression. Int. J. Mol. Sci. 21 (23), 9234. doi: 10.3390/ijms21239234 PMC7730936 33287416

[B10] Babinská K. Pivovarčiová A. Filčíková D. Tomova A. Ostatníková D. (2022). Association of Conduct Problems and Gastrointestinal Symptoms in Individuals With Autism Spectrum Disorders. Activitas Nervosa Superior Rediviva 58 (3), 69–72

[B11] Bik E. (2016). The Hoops, Hopes, and Hypes of Human Microbiome Research. Yale J. Biol. Med 89(3), 363–73. 27698620 PMC5045145

[B12] Bolte E. (1998). Autism and Clostridium Tetani. Med. Hypotheses 51 (2), 133–144. doi: 10.1016/s0306-9877(98)90107-4 9881820

[B13] Botta P. Fushiki A. Vicente A. Hammond L. Mosberger A. Gerfen C.. (2020). An Amygdala Circuit Mediates Experience-Dependent Momentary Arrests During Exploration. Cell. 183 (3), 605–619.e22. doi: 10.1016/j.cell.2020.09.023 33031743 PMC8276519

[B14] Bourassa M. Alim I. Bultman S. Ratan R. (2016). Butyrate, Neuroepigenetics and the Gut Microbiome: Can a High Fiber Diet Improve Brain Health? Neurosci. Lett. 625, 56–63. doi: 10.1016/j.neulet.2016.02.009 26868600 PMC4903954

[B15] Buffington S. Di Prisco G. Auchtung T. Ajami N. Petrosino J. Costa-Mattioli M. (2016). Microbial Reconstitution Reverses Maternal Diet-Induced Social and Synaptic Deficits in Offspring. Cell. 165 (7), 1762–1775. doi: 10.1016/j.cell.2016.06.001 27315483 PMC5102250

[B16] Cani P. Amar J. Iglesias M. Poggi M. Knauf C. Bastelica D.. (2007). Metabolic Endotoxemia Initiates Obesity and Insulin Resistance. Diabetes 56 (7), 1761–1772. doi: 10.2337/db06-1491 17456850

[B17] Carding S. Verbeke K. Vipond D. Corfe B. Owen L. (2015). Dysbiosis of the Gut Microbiota in Disease. Microbial Ecol. In Health Dis. 26 (0), 26191. doi: 10.3402/mehd.v26.26191 PMC4315779 25651997

[B18] Cenit M. Sanz Y. Codoñer-Franch P. (2017). Influence of Gut Microbiota on Neuropsychiatric Disorders. World J. Gastroenterol. 23(30), 5486–98. doi: 10.3748/wjg.v23.i30.5486 28852308 PMC5558112

[B19] Chauhan N. (2017). Human Gut Microbiome: An Imperative Element for Human Survival. Curr. Trends In Biomed. Eng. Biosci. 6 (1). doi: 10.19080/ctbeb.2017.06.555680

[B20] Chauhan N. Nain S. Sharma R. (2017). Identification of Arsenic Resistance Genes From Marine Sediment Metagenome. Indian J. Microbiol. 57 (3), 299–306. doi: 10.1007/s12088-017-0658-0 28904414 PMC5574776

[B21] Chen G. Chiang W. Shu B. Guo Y. Chiou S. Chiang T. (2017). Associations of Caesarean Delivery and the Occurrence of Neurodevelopmental Disorders, Asthma or Obesity in Childhood Based on Taiwan Birth Cohort Study. BMJ Open 7 (9), e017086. doi: 10.1136/bmjopen-2017-017086 PMC5623585 28963295

[B22] Chen Y. Zhao X. Moeder W. Tun H. Simons E. Mandhane P.. (2021). Impact of Maternal Intrapartum Antibiotics, and Caesarean Section With and Without Labour on Bifidobacterium and Other Infant Gut Microbiota. Microorganisms 9 (9), 1847. doi: 10.3390/microorganisms9091847 34576741 PMC8467529

[B23] Collado M. Rautava S. Aakko J. Isolauri E. Salminen S. (2016). Human Gut Colonisation may be Initiated *In Utero* by Distinct Microbial Communities in the Placenta and Amniotic Fluid. Sci. Rep. 6 (1), 23129. doi: 10.1038/srep23129 27001291 PMC4802384

[B24] Conlon M. Bird A. (2014). The Impact of Diet and Lifestyle on Gut Microbiota and Human Health. Nutrients 7 (1), 17–44. doi: 10.3390/nu7010017 25545101 PMC4303825

[B25] Cryan J. Dinan T. (2012). Mind-Altering Microorganisms: The Impact of the Gut Microbiota on Brain and Behaviour. Nat. Rev. Neurosci. 13 (10), 701–712. doi: 10.1038/nrn3346 22968153

[B26] Curran E. O’Neill S. Cryan J. Kenny L. Dinan T. Khashan A.. (2014). Research Review: Birth by Caesarean Section and Development of Autism Spectrum Disorder and Attention-Deficit/Hyperactivity Disorder: A Systematic Review and Meta-Analysis. J. Child Psychol. And Psychiatry 56 (5), 500–508. doi: 10.1111/jcpp.12351 25348074

[B27] de Bartolomeis A. Tomasetti C. Iasevoli F. (2015). Update on the Mechanism of Action of Aripiprazole: Translational Insights Into Antipsychotic Strategies Beyond Dopamine Receptor Antagonism. CNS Drugs 29 (9), 773–799. doi: 10.1007/s40263-015-0278-3 26346901 PMC4602118

[B28] DeFilippis M. Wagner K. D. (2016). Treatment of Autism Spectrum Disorder in Children and Adolescents. Psychopharmacol. Bull. 46 (2), 18–41. 27738378 10.64719/pb.4346 PMC5044466

[B29] De Filippo C. Cavalieri D. Di Paola M. Ramazzotti M. Poullet J. Massart S.. (2010). Impact of Diet in Shaping Gut Microbiota Revealed by a Comparative Study in Children From Europe and Rural Africa. Proc. Natl. Acad. Sci. 107 (33), 14691–14696. doi: 10.1073/pnas.1005963107 20679230 PMC2930426

[B30] Desbonnet L. Garrett L. Clarke G. Bienenstock J. Dinan T. (2008). The Probiotic Bifidobacterium Infantis: An Assessment of Potential Antidepressant Properties in the Rat. J. Psychiatr. Res. 43 (2), 164–174. doi: 10.1016/j.jpsychires.2008.03.009 18456279

[B31] Devika N. Raman K. (2019). Deciphering the Metabolic Capabilities of Bifidobacteria Using Genome-Scale Metabolic Models. Sci. Rep. 9 (1), 18222. doi: 10.1038/s41598-019-54696-9 31796826 PMC6890778

[B32] Dinan T. Stilling R. Stanton C. Cryan J. (2015). Collective Unconscious: How Gut Microbes Shape Human Behavior. J. Psychiatr. Res. 63, 1–9. doi: 10.1016/j.jpsychires.2015.02.021 25772005

[B33] Ding H. Taur Y. Walkup J. (2016). Gut Microbiota and Autism: Key Concepts and Findings. J. Autism And Dev. Disord. 47 (2), 480–489. doi: 10.1007/s10803-016-2960-9 27882443

[B34] Dogra S. Sakwinska O. Soh S. Ngom-Bru C. Brück W. Berger B.. (2015). Dynamics of Infant Gut Microbiota Are Influenced by Delivery Mode and Gestational Duration and Are Associated With Subsequent Adiposity. Mbio 6 (1), e02419–14. doi: 10.1128/mbio.02419-14 25650398 PMC4323417

[B35] Dominguez-Bello M. Costello E. Contreras M. Magris M. Hidalgo G. Fierer N.. (2010). Delivery Mode Shapes the Acquisition and Structure of the Initial Microbiota Across Multiple Body Habitats in Newborns. Proc. Of Natl. Acad. Of Sci. 107 (26), 11971–11975. doi: 10.1073/pnas.1002601107 20566857 PMC2900693

[B36] Eshraghi R. Deth R. Mittal R. Aranke M. Kay S. Moshiree B.. (2018). Early Disruption of the Microbiome Leading to Decreased Antioxidant Capacity and Epigenetic Changes: Implications for the Rise in Autism. Front Cell Neurosc 12, 256. doi: 10.3389/fncel.2018.00256 PMC6104136 30158857

[B37] Flannery J. Callaghan B. Sharpton T. Fisher P. Pfeifer J. (2019). Is Adolescence the Missing Developmental Link in Microbiome-Gut-Brain Axis Communication? Dev. Psychobiol. 61 (5), 783–795. doi: 10.1002/dev.21821 30690712 PMC6776431

[B38] Forssberg H. (2019). Microbiome Programming of Brain Development: Implications for Neurodevelopmental Disorders. Dev. Med. Child Neurol. 61 (7), 744–749. doi: 10.1111/dmcn.14208 30868564

[B39] Gershon M. (1999). The Enteric Nervous System: A Second Brain. Hosp. Pract. 34 (7), 31–52. doi: 10.3810/hp.1999.07.153 10418549

[B40] Gillor O. Etzion A. Riley M. (2008). The Dual Role of Bacteriocins as Anti- and Probiotics. Appl. Microbiol. And Biotechnol. 81 (4), 591–606. doi: 10.1007/s00253-008-1726-5 18853155 PMC2670069

[B41] Giri R. Sharma R. (2022). Analysis of Protein Association Networks Regulating the Neuroactive Metabolites Production in Lactobacillus Species. Enzyme Microbial Technol. 154, 109978. doi: 10.1016/j.enzmictec.2021.109978 34968825

[B42] Gondalia S. Palombo E. Knowles S. Cox S. Meyer D. Austin D. (2012). Molecular Characterisation of Gastrointestinal Microbiota of Children With Autism (With and Without Gastrointestinal Dysfunction) and Their Neurotypical Siblings. Autism Res. 5 (6), 419–427. doi: 10.1002/aur.1253 22997101

[B43] Goyal R. Hirano I. (1996). The Enteric Nervous System. N. Engl. J. Med. 334 (17), 1106–1115. doi: 10.1056/nejm199604253341707 8598871

[B44] Groer M. Gregory K. Louis-Jacques A. Thibeau S. Walker W. (2015). The Very Low Birth Weight Infant Microbiome and Childhood Health. Birth Defects Res. Part C: Embryo Today: Rev. 105 (4), 252–264. doi: 10.1002/bdrc.21115 26663857

[B45] Hesla H. Stenius F. Jäderlund L. Nelson R. Engstrand L. Alm J.. (2014). Impact of Lifestyle on the Gut Microbiota of Healthy Infants and Their Mothers - the ALADDIN Birth Cohort. FEMS Microbiol. Ecol. 90 (3), 791–801. doi: 10.1111/1574-6941.12434 25290507

[B46] Ikeyama N. Murakami T. Toyoda A. Mori H. Iino T. Ohkuma M.. (2020). Microbial Interaction Between the Succinate-Utilizing Bacterium Phascolarctobacterium Faecium and the Gut Commensal Bacteroides Thetaiotaomicron. Microbiologyopen 9 (10), e1111. doi: 10.1002/mbo3.1111 32856395 PMC7568257

[B47] Iljazovic A. Roy U. Gálvez E. Lesker T. Zhao B. Gronow A.. (2020). Perturbation of the Gut Microbiome by Prevotella Spp. Enhances Host Susceptibility to Mucosal Inflammation. Mucosal Immunol. 14 (1), 113–124. doi: 10.1038/s41385-020-0296-4 32433514 PMC7790746

[B48] Jašarević E. Howard C. Morrison K. Misic A. Weinkopff T. Scott P.. (2018). The Maternal Vaginal Microbiome Partially Mediates the Effects of Prenatal Stress on Offspring Gut and Hypothalamus. Nat. Neurosci. 21 (8), 1061–1071. doi: 10.1038/s41593-018-0182-5 29988069

[B49] Javier Díaz-García F. Flores-Medina S. Mercedes Soriano-Becerril D. (2020). Interplay Between Human Intestinal Microbiota and Gut-To-Brain Axis: Relationship With Autism Spectrum Disorders. Microorganisms., 318. doi: 10.5772/intechopen.89998

[B50] Jennis M. Cavanaugh C. Leo G. Mabus J. Lenhard J. Hornby P. (2017). Microbiota-Derived Tryptophan Indoles Increase After Gastric Bypass Surgery and Reduce Intestinal Permeability *In Vitro* and *In Vivo* . Neurogastroenterol.& Motil. 30 (2), e13178. doi: 10.1111/nmo.13178 28782205

[B51] Jernberg C. Löfmark S. Edlund C. Jansson J. (2007). Long-Term Ecological Impacts of Antibiotic Administration on the Human Intestinal Microbiota. ISME J. 1 (1), 56–66. doi: 10.1038/ismej.2007.3 18043614

[B52] Johnson A. Houtti M. Saboe A. Koecher K. Menon R. Knights D. (2021). Whole Wheat and Bran Cereal Affects Microbiome Stability. Curr. Developments In Nutr. 5 (Supplement_2), 1162–1162. doi: 10.1093/cdn/nzab054_017

[B53] Kabeerdoss J. Ferdous S. Balamurugan R. Mechenro J. Vidya R. Santhanam S.. (2013). Development of the Gut Microbiota in Southern Indian Infants From Birth to 6 Months: A Molecular Analysis. J. Nutr. Sci. 3, 5821. doi: 10.1017/jns.2013.6 PMC4153310 25191566

[B54] Kalnins G. Kuka J. Grinberga S. Makrecka-Kuka M. Liepinsh E. Dambrova M.. (2015). Structure and Function of CutC Choline Lyase From Human Microbiota Bacterium Klebsiella Pneumoniae. J. Biol. Chem. 290 (35), 21732–21740. doi: 10.1074/jbc.m115.670471 26187464 PMC4571895

[B55] Kang D. Adams J. Coleman D. Pollard E. Maldonado J. McDonough-Means S.. (2019). Long-Term Benefit of Microbiota Transfer Therapy on Autism Symptoms and Gut Microbiota. Sci. Rep. 9 (1), 10. doi: 10.1038/s41598-019-42183-0 30967657 PMC6456593

[B56] Kang D. Adams J. Gregory A. Borody T. Chittick L. Fasano A.. (2017). Microbiota Transfer Therapy Alters Gut Ecosystem and Improves Gastrointestinal and Autism Symptoms: An Open-Label Study. Microbiome 5 (1), e00413–20. doi: 10.1186/s40168-016-0225-7 PMC5264285 28122648

[B57] Kim G. Bae J. Kim M. Kwon H. Park G. Kim S.. (2020). Delayed Establishment of Gut Microbiota in Infants Delivered by Cesarean Section. Front. In Microbiol. 11. doi: 10.3389/fmicb.2020.02099 PMC7516058 33013766

[B58] Kim S. Kim H. Yim Y. Ha S. Atarashi K. Tan T.. (2017). Maternal Gut Bacteria Promote Neurodevelopmental Abnormalities in Mouse Offspring. Nature 549 (7673), 528–532. doi: 10.1038/nature23910 28902840 PMC5870873

[B59] Kim N. Yun M. Oh Y. Choi H. (2018). Mind-Altering With the Gut: Modulation of the Gut-Brain Axis With Probiotics. J. Microbiol. 56 (3), 172–182. doi: 10.1007/s12275-018-8032-4 29492874

[B60] Kivenson V. Giovannoni S. (2020). An Expanded Genetic Code Enables Trimethylamine Metabolism in Human Gut Bacteria. Msystems 5 (5), 10. doi: 10.1128/msystems.00413-20 PMC7593587 33109749

[B61] Krajmalnik-Brown R. Lozupone C. Kang D. Adams J. (2015). Gut Bacteria in Children With Autism Spectrum Disorders: Challenges and Promise of Studying How a Complex Community Influences a Complex Disease. Microbial Ecol. In Health Dis. 26 (0), 310. doi: 10.3402/mehd.v26.26914 PMC4359272 25769266

[B62] Kumar J. Kumar M. Chauhan N. (2015). Oceanobacillussp HM6, a Novel Glutenase Positive Microbe Isolated From Human Gut. J. Biotechnol. And Biomaterial 5 (6), 88.

[B63] Kumar J. Kumar M. Pandey R. Chauhan N. (2017). Physiopathology and Management of Gluten-Induced Celiac Disease. J. Food Sci. 82 (2), 270–277. doi: 10.1111/1750-3841.13612 28140462

[B64] Kumar Mondal A. Kumar J. Pandey R. Gupta S. Kumar M. Bansal G.. (2017). Comparative Genomics of Host–Symbiont and Free-Living Oceanobacillus Species. Genome Biol. Evol. 9 (5), 1175–1182. doi: 10.1093/gbe/evx076 28460092 PMC5425236

[B65] Kumar T. Pandey R. Chauhan N. (2020). Hypoxia Inducible Factor-1α: The Curator of Gut Homeostasis. Front. Cell. Infection Microbiol. 10. doi: 10.3389/fcimb.2020.00227 PMC7242652 32500042

[B66] Kumar J. Verma M. Kumar T. Gupta S. Pandey R. Yadav M.. (2018). S9A Serine Protease Engender Antigenic Gluten Catabolic Competence to the Human Gut Microbe. Indian J. Microbiol. 58 (3), 294–300. doi: 10.1007/s12088-018-0732-2 30013273 PMC6023808

[B67] Łaniewski P. Herbst-Kralovetz M. (2021). Bacterial Vaginosis and Health-Associated Bacteria Modulate the Immunometabolic Landscape in a 3D Model of Human Cervix. NPJ Biofilms Microbiomes 7 (1), eaah6500. doi: 10.1038/s41522-021-00259-8 PMC8669023 34903740

[B68] Lax S. Sangwan N. Smith D. Larsen P. Handley K. Richardson M.. (2017). Bacterial Colonization and Succession in a Newly Opened Hospital. Sci. Trans. Med. 9 (391), 3. doi: 10.1126/scitranslmed.aah6500 PMC5706123 28539477

[B69] Lee H. (2019). The Interaction Between Gut Microbiome and Nutrients on Development of Human Disease Through Epigenetic Mechanisms. Genomics Inf. 17 (3), e24. doi: 10.5808/gi.2019.17.3.e24 PMC6808642 31610620

[B70] Levy S. Mandell D. Merhar S. Ittenbach R. Pinto-Martin J. (2003). Use of Complementary and Alternative Medicine Among Children Recently Diagnosed With Autistic Spectrum Disorder. J. Dev. Behav. Pediatr. 24 (6), 418–423. doi: 10.1097/00004703-200312000-00003 14671475

[B71] Liang D. Leung R. Guan W. Au W. (2019). Correction to: Involvement of Gut Microbiome in Human Health and Disease: Brief Overview, Knowledge Gaps and Research Opportunities. Gut Pathog. 11 (1), 43. doi: 10.1186/s13099-019-0339-0 31832105 PMC6869277

[B72] Li Q. Han Y. Dy A. Hagerman R. (2017). The Gut Microbiota and Autism Spectrum Disorders. Front. Cell. Neurosci. 11. doi: 10.3389/fncel.2017.00120 PMC5408485 28503135

[B73] Liu F. Li J. Wu F. Zheng H. Peng Q. Zhou H. (2019). Altered Composition and Function of Intestinal Microbiota in Autism Spectrum Disorders: A Systematic Review. Trans. Psychiatry 9 (1), 10. doi: 10.1038/s41398-019-0389-6 PMC6351640 30696816

[B74] Li N. Yang J. Zhang J. Liang C. Wang Y. Chen B.. (2019). Correlation of Gut Microbiome Between ASD Children and Mothers and Potential Biomarkers for Risk Assessment. Genomics Proteomics Bioinf. 17 (1), 26–38. doi: 10.1016/j.gpb.2019.01.002 PMC6520911 31026579

[B75] Louis P. (2012). Does the Human Gut Microbiota Contribute to the Etiology of Autism Spectrum Disorders? Digestive Dis. And Sci. 57 (8), 1987–1989. doi: 10.1007/s10620-012-2286-1 22736019

[B76] Luna R. Savidge T. Williams K. (2016). The Brain-Gut-Microbiome Axis: What Role Does it Play in Autism Spectrum Disorder? Curr. Dev. Disord. Rep. 3 (1), 75–81. doi: 10.1007/s40474-016-0077-7 27398286 PMC4933016

[B77] MacFabe D. (2012). Short-Chain Fatty Acid Fermentation Products of the Gut Microbiome: Implications in Autism Spectrum Disorders. Microbial Ecol. Health Dis. 23 (0), 10. doi: 10.3402/mehd.v23i0.19260 PMC3747729 23990817

[B78] MacFabe D. (2015). Enteric Short-Chain Fatty Acids: Microbial Messengers of Metabolism, Mitochondria, and Mind: Implications in Autism Spectrum Disorders. Microbial Ecol. Health Dis. 26 (0), 3–7. doi: 10.3402/mehd.v26.28177 PMC4451098 26031685

[B79] Macfabe D. Cain D. Rodriguezcapote K. Franklin A. Hoffman J. Boon F.. (2007). Neurobiological Effects of Intraventricular Propionic Acid in Rats: Possible Role of Short Chain Fatty Acids on the Pathogenesis and Characteristics of Autism Spectrum Disorders. Behav. Brain Res. 176 (1), 149–169. doi: 10.1016/j.bbr.2006.07.025 16950524

[B80] MacFabe D. Rodriguez-K. Hoffman J. Franklin A. Mohammad-A Y. Taylor A.. (2008). A Novel Rodent Model of Autism: Intraventricular Infusions of Propionic Acid Increase Locomotor Activity and Induce Neuroinflammation and Oxidative Stress in Discrete Regions of Adult Rat Brain. Am. J. Biochem. Biotechnol. 4 (2), 146–166. doi: 10.3844/ajbbsp.2008.146.166

[B81] Macfarlane G. Cummings J. Allison C. (1986). Protein Degradation by Human Intestinal Bacteria. Microbiology 132 (6), 1647–1656. doi: 10.1099/00221287-132-6-1647 3543210

[B82] Ma B. Liang J. Dai M. Wang J. Luo J. Zhang Z.. (2019). Altered Gut Microbiota in Chinese Children With Autism Spectrum Disorders. Front. In Cell. Infection Microbiol. 9. doi: 10.3389/fcimb.2019.00040 PMC6414714 30895172

[B83] Mangiola F. (2016). Gut Microbiota in Autism and Mood Disorders. World J. Gastroenterol. 22 (1), 361. doi: 10.3748/wjg.v22.i1.361 26755882 PMC4698498

[B84] Matsuzaki H. Iwata K. Manabe T. Mori N. (2012). Triggers for Autism: Genetic and Environmental Factors. J. Cent. Nervous System Dis. 4, JCNSD.S9058. doi: 10.4137/jcnsd.s9058 PMC3619552 23650465

[B85] Mayer E. Knight R. Mazmanian S. Cryan J. Tillisch K. (2014). Gut Microbes and the Brain: Paradigm Shift in Neuroscience. J. Neurosci. 34 (46), 15490–15496. doi: 10.1523/jneurosci.3299-14.2014 25392516 PMC4228144

[B86] Mimee M. Citorik R. Lu T. (2016). Microbiome Therapeutics — Advances and Challenges. Advanced Drug Deliv. Rev. 105, 44–54. doi: 10.1016/j.addr.2016.04.032 PMC5093770 27158095

[B87] Mitsou E. Kirtzalidou E. Oikonomou I. Liosis G. Kyriacou A. (2008). Fecal Microflora of Greek Healthy Neonates. Anaerobe 14 (2), 94–101. doi: 10.1016/j.anaerobe.2007.11.002 18207437

[B88] Mohammad-Zadeh L. Moses L. Gwaltney-Brant S. (2008). Serotonin: A Review. J. Veterinary Pharmacol. Ther. 31 (3), 187–199. doi: 10.1111/j.1365-2885.2008.00944.x 18471139

[B89] Molnár Z. Garel S. López-Bendito G. Maness P. Price D. (2012). Mechanisms Controlling the Guidance of Thalamocortical Axons Through the Embryonic Forebrain. Eur. J. Neurosci. 35 (10), 1573–1585. doi: 10.1111/j.1460-9568.2012.08119.x 22607003 PMC4370206

[B90] Monda V. Villano I. Messina A. Valenzano A. Esposito T. Moscatelli F.. (2017). Exercise Modifies the Gut Microbiota With Positive Health Effects. Oxid. Med. Cell. Longevity 2017, 1–8. doi: 10.1155/2017/3831972 PMC5357536 28357027

[B91] Nitschke A. Deonandan R. Konkle A. (2020). The Link Between Autism Spectrum Disorder and Gut Microbiota: A Scoping Review. Autism 24 (6), 1328–1344. doi: 10.1177/1362361320913364 32340474

[B92] Ni J. Wu G. Albenberg L. Tomov V. (2017). Gut Microbiota and IBD: Causation or Correlation? Nat. Rev. Gastroenterol. Hepatol. 14 (10), 573–584. doi: 10.1038/nrgastro.2017.88 28743984 PMC5880536

[B93] Noor R. Maniha S. Taniya M. (2020). Cesarean Section Delivery and the Autism Spectrum Disorder: Risk and Consequences in Bangladesh. Biomed. Biotechnol. Res. J. 4 (1), 2115. doi: 10.4103/bbrj.bbrj_134_19

[B94] Noor R. Naz A. Maniha S. Tabassum N. Tabassum T. Tabassum T.. (2021). Microorganisms and Cardiovascular Diseases: Importance of Gut Bacteria. Front. In Bioscience-Landmark 26 (5), 22. doi: 10.52586/4921 34027647

[B95] O’Mahony S. Marchesi J. Scully P. Codling C. Ceolho A. Quigley E.. (2009). Early Life Stress Alters Behavior, Immunity, and Microbiota in Rats: Implications for Irritable Bowel Syndrome and Psychiatric Illnesses. Biol. Psychiatry 65 (3), 263–267. doi: 10.1016/j.biopsych.2008.06.026z 18723164

[B96] Oriach C. Robertson R. Stanton C. Cryan J. Dinan T. (2016). Food for Thought: The Role of Nutrition in the Microbiota-Gut–Brain Axis. Clin. Nutr. Exp. 6, 25–38. doi: 10.1016/j.yclnex.2016.01.003

[B97] Palmer C. Bik E. DiGiulio D. Relman D. Brown P. (2007). Development of the Human Infant Intestinal Microbiota. PLoS Biol. 5 (7), e177. doi: 10.1371/journal.pbio.0050177 17594176 PMC1896187

[B98] Pandey P. Verma P. Kumar H. Bavdekar A. Patole M. Shouche Y. (2012). Comparative Analysis of Fecal Microflora of Healthy Full-Term Indian Infants Born With Different Methods of Delivery (Vaginal vs Cesarean): Acinetobacter Sp. Prevalence in Vaginally Born Infants. J. Biosci. 37 (S1), 989–998. doi: 10.1007/s12038-012-9268-5 23151789

[B99] Parracho H. Bingham M. Gibson G. McCartney A. (2005). Differences Between the Gut Microflora of Children With Autistic Spectrum Disorders and That of Healthy Children. J. Med. Microbiol. 54 (10), 987–991. doi: 10.1099/jmm.0.46101-0 16157555

[B100] Penders J. Thijs C. Vink C. Stelma F. Snijders B. Kummeling I.. (2006). Factors Influencing the Composition of the Intestinal Microbiota in Early Infancy. Pediatrics 118 (2), 511–521. doi: 10.1542/peds.2005-2824 16882802

[B101] Penders J. Vink C. Driessen C. London N. Thijs C. tobberingh E. (2005). Quantification Ofbifidobacteriumspp., *Escherichia Coli* and Clostridiumdifficileifaecal Samples of Breast-Fed and Formula-Fed Infants by Real-Time PCR. FEMS Microbiol. Lett. 243 (1), 141–147. doi: 10.1016/j.femsle.2004.11.052 15668012

[B102] Rosenfeld C. (2015). Microbiome Disturbances and Autism Spectrum Disorders. Drug Metab. Disposition 43 (10), 1557–1571. doi: 10.1124/dmd.115.063826 25852213

[B103] Sanders M. Guarner F. Guerrant R. Holt P. Quigley E. Sartor R.. (2013). An Update on the Use and Investigation of Probiotics in Health and Disease. Gut 62 (5), 787–796. doi: 10.1136/gutjnl-2012-302504 23474420 PMC4351195

[B104] Sandler R. Finegold S. Bolte E. Buchanan C. Maxwell A. Väisänen M.. (2000). Short-Term Benefit From Oral Vancomycin Treatment of Regressive-Onset Autism. J. Child Neurol. 15 (7), 429–435. doi: 10.1177/088307380001500701 10921511

[B105] Scott K. Gratz S. Sheridan P. Flint H. Duncan S. (2012) The Influence of Diet on the Gut Microbiota (Accessed 29 October 2021). 10.1016/j.phrs.2012.10.020 23147033

[B106] Sealy L. (1978). The Effect of Sodium Butyrate on Histone Modification. Cell 14 (1), 115–121. doi: 10.1016/0092-8674(78)90306-9 667928

[B107] Shao Y. Forster S. Tsaliki E. Vervier K. Strang A. Simpson N.. (2019). Stunted Microbiota and Opportunistic Pathogen Colonization in Caesarean-Section Birth. Nature 574 (7776), 117–121. doi: 10.1038/s41586-019-1560-1 31534227 PMC6894937

[B108] Shapiro D. Renock S. Arrington E. Chiodo L. Liu L. Sibley D.. (2003). Aripiprazole, A Novel Atypical Antipsychotic Drug With a Unique and Robust Pharmacology. Neuropsychopharmacology 28 (8), 1400–1411. doi: 10.1038/sj.npp.1300203 12784105

[B109] Sharon G. Cruz N. Kang D. Gandal M. Wang B. Kim Y.. (2019). Human Gut Microbiota From Autism Spectrum Disorder Promote Behavioral Symptoms in Mice. Cell 177 (6), 1600–1618.e17. doi: 10.1016/j.cell.2019.05.004 31150625 PMC6993574

[B110] Shaw W. (2010). Increased Urinary Excretion of a 3-(3-Hydroxyphenyl)-3-Hydroxypropionic Acid (HPHPA), an Abnormal Phenylalanine Metabolite Ofclostridiaspp. In the Gastrointestinal Tract, in Urine Samples From Patients With Autism and Schizophrenia. Nutr. Neurosci. 13 (3), 135–143. doi: 10.1179/147683010x12611460763968 20423563

[B111] Shen H. (2015). Microbes on the Mind. Proc. Natl. Acad. Of Sci. 112 (30), 9143–9145. doi: 10.1073/pnas.1509590112 26221006 PMC4522824

[B112] Sleiman S. Basso M. Mahishi L. Kozikowski A. Donohoe M. Langley B.. (2009). Putting the ‘Hat’ Back on Survival Signalling: The Promises and Challenges of HDAC Inhibition in the Treatment of Neurological Conditions. Expert Opin. Investigational Drugs 18 (5), 573–584. doi: 10.1517/13543780902810345 PMC2731419 19388875

[B113] Soorya L. Kiarashi J. Hollander E. (2008). Psychopharmacologic Interventions for Repetitive Behaviors in Autism Spectrum Disorders. Child And Adolesc. Psychiatr. Clinics Of North America 17 (4), 753–771. doi: 10.1016/j.chc.2008.06.003 18775368

[B114] Srikantha P. Mohajeri M. (2019). The Possible Role of the Microbiota-Gut-Brain-Axis in Autism Spectrum Disorder. Int. J. Of Mol. Sci. 20 (9) 2115. doi: 10.3390/ijms20092115 PMC6539237 31035684

[B115] Stewart C. Skeath T. Nelson A. Fernstad S. Marrs E. Perry J.. (2015). Preterm Gut Microbiota and Metabolome Following Discharge From Intensive Care. Sci. Rep. 5 (1), 17141. doi: 10.1038/srep17141 26598071 PMC4657104

[B116] Stout M. Conlon B. Landeau M. Lee I. Bower C. Zhao Q.. (2013). Identification of Intracellular Bacteria in the Basal Plate of the Human Placenta in Term and Preterm Gestations. Am. J. Obstetrics Gynecol. 208 (3), 226.e1–226.e7. doi: 10.1016/j.ajog.2013.01.018 PMC3740162 23333552

[B117] Sun B. Hou L. Yang Y. (2020). Effects of Eubiotic Lignocellulose on the Gut Microbiota and Metabolism of Chickens. Front. Veterinary Sci. 8, 668003. doi: 10.21203/rs.3.rs-49629/v1 PMC8473647 34589531

[B118] Taddei C. Cortez R. Mattar R. Torloni M. Daher S. (2018). Microbiome in Normal and Pathological Pregnancies: A Literature Overview. Am. J. Reprod. Immunol. 80 (2), e12993. doi: 10.1111/aji.12993 29873429

[B119] Toscano M. De Grandi R. Peroni D. Grossi E. Facchin V. Comberiati P.. (2017). Impact of Delivery Mode on the Colostrum Microbiota Composition. BMC Microbiol. 17 (1), 205. doi: 10.1186/s12866-017-1109-0 28946864 PMC5613475

[B120] Tsai L. (2000). Children With Autism Spectrum Disorder. Focus On Autism And Other Dev. Disabil. 15 (3), 138–145. doi: 10.1177/108835760001500302

[B121] Tsai P. Hull C. Chu Y. Greene-Colozzi E. Sadowski A. Leech J.. (2012). Autistic-Like Behaviour and Cerebellar Dysfunction in Purkinje Cell Tsc1 Mutant Mice. Nature 488 (7413), 647–651. doi: 10.1038/nature11310 22763451 PMC3615424

[B122] Ubeda C. Taur Y. Jenq R. Equinda M. Son T. Samstein M.. (2010). Vancomycin-Resistant Enterococcus Domination of Intestinal Microbiota is Enabled by Antibiotic Treatment in Mice and Precedes Bloodstream Invasion in Humans. J. Clin. Invest. 120 (12), 4332–4341. doi: 10.1172/jci43918 21099116 PMC2993598

[B123] Umbrello G. Esposito S. (2016). Microbiota and Neurologic Diseases: Potential Effects of Probiotics. J. Trans. Med. 14 (1), 298. doi: 10.1186/s12967-016-1058-7 PMC5069982 27756430

[B124] Valicenti-McDermott M. McVicar K. Rapin I. Wershil B. Cohen H. Shinnar S. (2006). Frequency of Gastrointestinal Symptoms in Children With Autistic Spectrum Disorders and Association With Family History of Autoimmune Disease. J. Dev. Behav. Pediatr. 27 (Supplement 2), S128–S136. doi: 10.1097/00004703-200604002-00011 16685179

[B125] Van De Sande M. van Buul V. Brouns F. (2014). Autism and Nutrition: The Role of the Gut–Brain Axis. Nutr. Res. Rev. 27 (2), 199–214. doi: 10.1017/s0954422414000110 25004237

[B126] Van Nimwegen F. Penders J. Stobberingh E. Postma D. Koppelman G. Kerkhof M.. (2011). Mode and Place of Delivery, Gastrointestinal Microbiota, and Their Influence on Asthma and Atopy. J. Allergy And Clin. Immunol. 128 (5), 948–955.e3. doi: 10.1016/j.jaci.2011.07.027 21872915

[B127] Vuong H. Pronovost G. Williams D. Coley E. Siegler E. Qiu A.. (2020). The Maternal Microbiome Modulates Fetal Neurodevelopment in Mice. Nature 586 (7828), 281–286. doi: 10.1038/s41586-020-2745-3 32968276 PMC7554197

[B128] Vuong H. Yano J. Fung T. Hsiao E. (2017). The Microbiome and Host Behavior. Annu. Rev. Neurosci. 40 (1), 21–49. doi: 10.1146/annurev-neuro-072116-031347 28301775 PMC6661159

[B129] Wang L. Christophersen C. Sorich M. Gerber J. Angley M. Conlon M. (2012). Elevated Fecal Short Chain Fatty Acid and Ammonia Concentrations in Children With Autism Spectrum Disorder. Digestive Dis. Sci. 57 (8), 2096–2102. doi: 10.1007/s10620-012-2167-7 22535281

[B130] Wang L. Conlon M. Christophersen C. Sorich M. Angley M. (2014). Gastrointestinal Microbiota and Metabolite Biomarkers in Children With Autism Spectrum Disorders. Biomarkers In Med. 8 (3), 331–344. doi: 10.2217/bmm.14.12 24712423

[B131] Wang W. Xu S. Ren Z. Tao L. Jiang J. Zheng S. (2015). Application of Metagenomics in the Human Gut Microbiome. World J. Gastroenterol. 21 (3), 803. doi: 10.3748/wjg.v21.i3.803 25624713 PMC4299332

[B132] Warner B. (2018). The Contribution of the Gut Microbiome to Neurodevelopment and Neuropsychiatric Disorders. Pediatr. Res. 85 (2), 216–224. doi: 10.1038/s41390-018-0191-9 30283047

[B133] Wikoff W. Anfora A. Liu J. Schultz P. Lesley S. Peters E.. (2009). Metabolomics Analysis Reveals Large Effects of Gut Microflora on Mammalian Blood Metabolites. Proc. Natl. Acad. Sci. 106 (10), 3698–3703. doi: 10.1073/pnas.0812874106 19234110 PMC2656143

[B134] Wong H. Smith R. (2006). Patterns of Complementary and Alternative Medical Therapy Use in Children Diagnosed With Autism Spectrum Disorders. J. Autism Dev. Disord. 36 (7), 901–909. doi: 10.1007/s10803-006-0131-0 16897395

[B135] Xu M. (2015). Fecal Microbiota Transplantation Broadening its Application Beyond Intestinal Disorders. World J. Gastroenterol. 21 (1), 102. doi: 10.3748/wjg.v21.i1.102 25574083 PMC4284325

[B136] Yadav M. Chauhan N. (2020). Overview of the Rules of the Microbial Engagement in the Gut Microbiome: A Step Towards Microbiome Therapeutics. J. Appl. Microbiol. 130 (5), 1425–1441. doi: 10.1111/jam.14883 33022786

[B137] Yadav M. Chauhan N. (2021). Microbiome Therapeutics: Exploring the Present Scenario and Challenges. Gastroenterol. Rep. 10, 1–19. doi: 10.1093/gastro/goab046 PMC8972995 35382166

[B138] Yadav M. Pandey R. Chauhan N. (2020). Catabolic Machinery of the Human Gut Microbes Bestow Resilience Against Vanillin Antimicrobial Nature. Front. Microbiol. 11. doi: 10.3389/fmicb.2020.588545 PMC7605359 33193247

[B139] Yadav M. Verma M. Chauhan N. (2017). A Review of Metabolic Potential of Human Gut Microbiome in Human Nutrition. Arch. Of Microbiol. 200 (2), 203–217. doi: 10.1007/s00203-017-1459-x 29188341

[B140] Yaguchi K. Nishimura-Akiyoshi S. Kuroki S. Onodera T. Itohara S. (2014). Identification of Transcriptional Regulatory Elements for Ntng1 and Ntng2 Genes in Mice. Mol. Brain 7 (1), 19. doi: 10.1186/1756-6606-7-19 24642214 PMC4000137

[B141] Yang L. Lu X. Nossa C. Francois F. Peek R. Pei Z. (2009). Inflammation and Intestinal Metaplasia of the Distal Esophagus Are Associated With Alterations in the Microbiome. Gastroenterology 137 (2), 588–597. doi: 10.1053/j.gastro.2009.04.046 19394334 PMC2963147

[B142] Yip B. Leonard H. Stock S. Stoltenberg C. Francis R. Gissler M.. (2016). Caesarean Section and Risk of Autism Across Gestational Age: A Multi-National Cohort Study of 5 Million Births. Int. J. Epidemiol. 46, 429–39. doi: 10.1093/ije/dyw336 PMC5837358 28017932

[B143] Zhu Q. Gao R. Wu W. Qin H. (2013). The Role of Gut Microbiota in the Pathogenesis of Colorectal Cancer. Tumor Biol. 34 (3), 1285–1300. doi: 10.1007/s13277-013-0684-4 23397545

